# Patient Satisfaction With a Coach-Guided, Technology-Based Mental Health Treatment: Qualitative Interview Study and Theme Analysis

**DOI:** 10.2196/50977

**Published:** 2024-02-02

**Authors:** Ashley Helm Smith, Hilary Touchett, Patricia Chen, Terri Fletcher, Jennifer Arney, Julianna Hogan, Miryam Wassef, Marylene Cloitre, Jan A Lindsay

**Affiliations:** 1 Houston Veteran Affairs Health Services Research and Development Center for Innovations in Quality, Effectiveness and Safety Michael E DeBakey Veteran Affairs Medical Center Houston, TX United States; 2 South Central Mental Illness Research, Education and Clinical Center Houston, TX United States; 3 Department of Medicine Baylor College of Medicine Houston, TX United States; 4 Menninger Department of Psychiatry and Behavioral Sciences Baylor College of Medicine Houston, TX United States; 5 Department of Sociology College of Human Sciences and Humanities University of Houston Clear Lake Houston, TX United States; 6 National Center for Post-Traumatic Stress Disorder Dissemination and Training Division Veteran Affairs Palo Alto Health Care System Palo Alto, CA United States; 7 Rice University’s Baker Institute for Public Policy Houston, TX United States

**Keywords:** coaching, digital treatment, interview, mental health, patient satisfaction, PTSD, qualitative assessment, qualitative methods, sentiment analysis, technology-based, telehealth, trauma, veterans, video telehealth, web-based treatment

## Abstract

**Background:**

Technology-based mental health interventions address barriers rural veterans face in accessing care, including provider scarcity and distance from the hospital or clinic. webSTAIR is a 10-module, web-based treatment based on Skills Training in Affective and Interpersonal Regulation, designed to treat posttraumatic stress disorder and depression in individuals exposed to trauma. Previous work has demonstrated that webSTAIR is acceptable to participants and effective at reducing symptoms of posttraumatic stress disorder and depression when delivered synchronously or asynchronously (over 5 or 10 sessions).

**Objective:**

This study explored factors that lead to greater patient satisfaction with webSTAIR, a web-based, coach-guided intervention.

**Methods:**

We analyzed qualitative interview data to identify themes related to patient satisfaction with webSTAIR delivered with synchronous video-based coaching.

**Results:**

Four themes emerged from the data: (1) coaching provides accountability and support, (2) self-pacing offers value that meets individual needs, (3) participants like the comfort and convenience of the web-based format, and (4) technical issues were common but not insurmountable.

**Conclusions:**

We conclude that participants valued the accountability, flexibility, and convenience of tech-based interventions with video-delivered coaching.

## Introduction

More than 1.7 million US veterans receive mental health care through the Veterans Health Administration (VHA) annually [1]. However, US veterans underuse mental health care and often experience barriers that impact access, such as provider scarcity [2], distance to clinic [3], inability to take off from work or school, and dependent care responsibilities [4-6]. Veterans living in rural areas make up about 24% of all veterans in the United States [7], and they have similar mental health care needs as urban veterans; however, rural veterans experience access barriers at higher rates [8,9]. Technology-based mental health interventions are a potential solution to address some of the barriers that rural veterans in the United States face.

Technology-based mental health interventions can be accessed from a smart phone or tablet. They range from fully automated to therapist-guided in person or by video, telephone, or SMS text messages [10-13]. Technology-based mental health interventions have demonstrated effectiveness in clinical trials, with moderate-to-high effect sizes [14-17]. However, satisfaction with a treatment plays a significant role in treatment effectiveness. Patient satisfaction with treatment is linked to greater adherence [18,19] and greater perceived improvement in clinical status [20,21]. Even when adherence to treatment is controlled for, satisfied patients benefit more from care than less satisfied patients [22,23]. In mental health settings, a strong therapeutic alliance is linked to greater patient satisfaction [24]. Further understanding of factors that contribute to patient satisfaction is critical to designing future tech-based interventions that demonstrate better engagement and retention and greater clinical benefit [25,26].

Studying satisfaction with mental health treatments is complex. There are a number of variables that can influence patient satisfaction with mental health treatment, such as patient, disease, provider, therapy, and environmental factors (eg, technology-mediated treatments) [22,27,28]. Although many teams evaluate overall satisfaction by collecting survey data and compiling ratings to quantify patients’ levels of satisfaction, qualitative data provides a nuanced and contextualized understanding of satisfaction [22]. In 2 recent studies, our team found that having therapist support and the convenience of being able to access treatment on the web contributed to patient satisfaction with technology-based mental health interventions [29,30]. This study uses qualitative interviews with patients who completed an internet-based mental health treatment to explore factors that lead to greater patient satisfaction in such a program. This study builds on previous findings by exploring the benefits of using a coach and the ways web-based formats provide comfort and convenience, as well as increased access in some cases.

## Methods

### Study Design

webSTAIR is an internet-based intervention adapted from Skills Training in Affective and Interpersonal Regulation (STAIR), a treatment for trauma-exposed individuals with symptoms of posttraumatic stress disorder (PTSD) and depression [31]. The program was developed to engage rural women veterans, who have been found to be underrepresented in mental health services [32]. However, male veterans were also enrolled if they were interested and met the study criteria. webSTAIR offers 10 web-based modules of self-directed skills training on emotion regulation (eg, emotional awareness, emotional management, and distress tolerance) and interpersonal skills (eg, assertiveness, flexibility, and compassion for oneself and others).

In addition to the asynchronously delivered web-based content, participants were offered 5 or 10 synchronous video coaching sessions, which took place using video-telehealth, after the completion of 1 or 2 modules. Coaches were licensed Veterans Affairs (VA) mental health providers who underwent training in the intervention and attended weekly supervision with a certified STAIR trainer. Sessions with the coach lasted approximately 45-50 minutes and involved reviewing module content, discussing the applicability of concepts in the veteran’s life, and strategizing on how to integrate skills into daily practice. The webSTAIR program was delivered as routine care in mental health outpatient clinics at 9 sites serving rural patients within the VHA. The qualitative data presented were collected as part of a naturalistic evaluation of the program.

### Recruitment and Sample

Participants in the webSTAIR program were US veterans recruited from 9 VHA facilities across the country that serve rural patients. Referrals were either clinician- or self-initiated. Participants completed the program between September 2018 and March 2020. Eligibility for the study was determined based on an initial telephone screening. Patients were considered eligible if they reported a history of trauma exposure and screened positive on the Primary Care PTSD Screen (PC-PTSD; positive on 3 of 5 items [33]) or the 2-item Patient Health Questionnaire (PHQ-2; positive on 1 of 2 items [34]), indicating clinically significant symptoms of PTSD or depression. The following resulted in ineligibility for enrollment: cognitive impairment or psychosis, mania, primary drug or alcohol abuse, current domestic violence, concurrent trauma-focused treatment, residential care for PTSD within 1 year, or an inability to attend telemental health appointments by video.

### Measures and Data Collection

Demographic information for all patients in the program was collected at the initial intake phase of the study. The qualitative data for this study were collected during a 1-time interview conducted following the completion of the webSTAIR program. Patients who completed the program were sent a letter explaining the purpose of the interview and study details. This letter also included a contact number that patients could call to decline participation. All patients who did not actively call to decline were then contacted by study staff and asked if they would like to participate. Study staff made at least 3 attempts to contact participants. Outreach resulted in a response rate of 30%, with 74 participants agreeing to participate. The demographic information of interviewees was linked using a study ID number.

The interview guide was piloted and refined during the pilot intervention phase of the study [30,35]. It contained both categorical and open-ended questions. One member of the study staff conducted the interview, while 1-2 staff members took detailed notes during the interview. While the study team did not record or transcribe interviews, note-takers were often able to capture responses nearly verbatim. Quotes in this paper are drawn from these notes.

Participants were asked questions about their experience working through the program and about their satisfaction with the intervention. Interviews were conducted by telephone by study staff, including the first author (AHS) and second author (HT), as well as other members of the study team (MW). Interviews lasted approximately 60 minutes. At the conclusion of the interview, the interview notes were consolidated, and the finalized interview notes were entered into a database. Interview protocols were reviewed by the principal investigator’s affiliated institutions’ institutional review board, designated as “quality improvement.”

### Data Analysis

Interview data were analyzed using strategies from qualitative content analysis and thematic analysis [36-39]. The interview notes for each participant were entered into a matrix in Microsoft Excel (Microsoft Corp) and organized by interview question. A list of the interview questions used in the analysis for this study can be found in Multimedia Appendix 1. Data coding and analysis were conducted by a master’s-level researcher with extensive qualitative experience who also functioned as a research assistant on the study (AHS) and a postdoctoral nurse trained in qualitative methods (HT). A codebook was developed based on interview guide questions and refined based on emergent findings. The second author (HT) reviewed the codes assigned by the first author, either agreeing or disagreeing. Coders met to resolve any disagreements until consensus was reached. The themes were analyzed and further defined to describe participants’ satisfaction with the intervention. The data are presented here by satisfaction with the web-based, coach-guided format of webSTAIR.

### Ethical Considerations

Study procedures were reviewed and found to be exempt by the institutional review board for Baylor College of Medicine and Affiliated Hospitals. Data were collected under a quality improvement designation. Participant confidentiality was upheld with the use of a randomized participant ID number.

## Results

### Sample

The exit interview was completed by 74 participants, the majority of whom were rural (46/74, 62%), White or Caucasian (48/74, 65%), female (42/74, 57%), aged between 35 and 44 years (25/74, 34%), and had some college or a 2-year college degree (32/74, 44%). A detailed list of participant characteristics can be found in Table 1.

**Table 1:** Participant demographics.

Demographics		Coach 5 (n=43), n (%)	Coach 10 (n=31), n (%)	Total (N=74), n (%)
**Sex**				
	Male	14 (33)	17 (55)	31 (42)
	Female	29 (67)	13 (42)	42 (57)
	Transgender	0 (0)	1 (3)	1 (1)
**Age (years)**				
	25-34	8 (19)	5 (16)	13 (18)
	35-44	16 (37)	9 (29)	25 (34)
	45-54	8 (19)	7 (23)	15 (20)
	55-64	9 (21)	7 (23)	16 (22)
	65-74	2 (4)	3 (9)	5 (6)
**Rurality**				
	Urban	16 (37)	12 (39)	28 (38)
	Rural	27 (63)	19 (61)	46 (62)
**Race**				
	White or Caucasian	24 (56)	24 (78)	48 (65)
	Black or African American	6 (14)	2 (6)	8 (11)
	Hispanic or Latino	4 (9)	2 (6)	6 (8)
	American Indian	1 (2)	1 (4)	2 (2)
	Mixed race or ethnicity	8 (19)	2 (6)	10 (14)
**Employment status**				
	Full-time	12 (28)	13 (42)	25 (34)
	Part-time	6 (13)	4 (13)	10 (13)
	Not currently working for pay	11 (26)	5 (16)	16 (22)
	Retired	14 (33)	9 (29)	23 (31)
**Educational level**				
	Some high school	1 (2)	0 (0)	1 (1)
	Earned high school degree	4 (9)	4 (13)	8 (11)
	Some college or 2-year degree	20 (47)	12 (39)	32 (44)
	Earned 4-year degree	14 (33)	10 (32)	24 (32)
	Postgraduate	3 (7)	5 (16)	8 (11)
	Missing	1 (2)	0 (0)	1 (1)

### Themes

Participants responded to categorical questions that they were generally satisfied with the webSTAIR program; that is, the majority of interview participants felt that webSTAIR met their needs and that they would use a similar program again in the future. Four themes emerged from the open-ended responses and are detailed below: (1) coaching provides accountability and support, (2) self-pacing offers value that meets individual needs, (3) participants like the comfort and convenience of the web-based format, and (4) technical issues were common but not insurmountable. Table 2 presents quotations exemplifying each theme.

**Table 2:** Domains and illustrative quotes.

Themes	Illustrative quotes
Theme 1: coaching provides accountability and support	Because she was so good at bringing back the tools you get out of webSTAIR. Website tells you to apply it, but it's nice to hear it differently and she brings it back to my situation. That was really helpful for me (35-44–year-old White female). Seemed like sessions were the practice/reiteration part. She really provided the application, tying it together and making it stick. Would stress this a lot. It really helped and challenged me. (35-44–year-old White male)
Theme 2: self-pacing offers value that meets individual needs	It allowed me to go at my own pace. I had time to consider my answers and think about what I wanted to say. (55-64–year-old White male) When you're meeting in person, you often are bobble heading even if you're not getting what they're telling you. Reading for yourself and being able to go over it and process… gives you time to process. (55-64–year-old mixed-race female) Some lessons needed more time to practice and to mentally digest. I'm still going over them because I printed them out, but I feel like I needed more time... (45-54–year-old White female)
Theme 3: participants like the comfort and convenience of the web-based format	I work, helped me not be stressed about getting appointments without taking off work. (25-34–year-old African American female) With PTSD, I don’t sleep well. To not have to get up and drive. Closest facility is 45 min-1 hour away…To not have to drive is great. I live in the country on purpose. I wouldn’t go to therapy without webSTAIR. (35-44–year-old mixed-race female) I liked that it was online and for whatever reason felt like a safer environment than sitting face to face with someone. (25-34–year-old White female) It's easy to get complacent too, catch 22 at your home. Easy to get distracted, too comfortable at home so easily distracted. (25-34–year-old White male)
Theme 4: technical issues are common but not insurmountable	If you're working on something and push the back button you delete everything you did. You have to start over. (35-44–year-old African American female) I didn't like the fact the info I wrote down in the modules, therapist couldn't see, so when we were reviewing, I would have to go back in and start from the beginning. It would be helpful if she could see what I wrote. (35-44–year-old African American female) Sometimes couldn't hear each other or it would freeze. Normal facetime issues. More issues with computer, iPad or iPhone crystal clear, perfect. (35-44–year-old American Indian or Alaskan Native female)

### Theme 1: Coaching Provides Accountability and Support

The presence of regular check-ins with a coach kept participants accountable and motivated them to do the work and stay on track by providing guidance as to when they should complete the next module. Only 1 participant noted that the regular check-ins with their coach and expectations about content completion each week felt like too much pressure, but this was uncommonly reported. Most respondents indicated they appreciated check-ins.

I knew that we were going to have our sessions. So it helped with accountability – like actually doing the work because we’re going to talk about it. And she keeps me on track because she tells me this is when you’re going to do the next module.

35-44-year-old Black or African-American female

Coaches provided emotional support and helped participants understand and apply the webSTAIR content to their own lives. Participants often noted a good rapport with their coach, and many would have liked to talk to them more simply because they enjoyed talking with them. As a mixed-race male in his early 30s commented, “She was awesome. She always remembered what was going on with me. She helped with materials.”

The majority of participants were satisfied with the number of sessions with their coach, regardless of the number of coaching sessions they received. Those that did express a preference for more sessions cited a variety of reasons, most commonly the desire for more emotional support and clarification about the content. Participants perceived coaching sessions as a time to process their feelings and better understand how content applied to their individual situation. The coaching sessions were considered an essential component of webSTAIR and were highly valued.

### Theme 2: Self-Pacing Offers Value That Meets Individual Needs

Participants generally liked the format of webSTAIR, which allowed them to take their time and interact with and respond to the web-based content. Having time between reviewing the material and meeting with their coach helped participants feel less pressure and less rushed than standard face-to-face psychotherapy using evidence-based psychotherapy protocols, as in “No pressure to feel like you get an answer right or wrong or in a hurry to get it done” (35-44–year-old White male). They were able to reflect on and practice the skills webSTAIR aimed to teach them. In many cases, this led to more thoughtful reactions to the content, which informed meetings with their coach and made them more productive. A White male in his mid-forties commented:

It made you use your mind and think about stuff. You can go to counseling all day long and you don’t get as much accomplished in an hour... [I] think on your own time, allows you to think outside the box.

35-44–year-old White male

In contrast to those who liked the self-paced format, a small number of participants reported they would have liked the pace to be more personalized or more self-paced. For example, a few participants would have liked to speed through some of the content that they felt they did not need or take more time (eg, more time in between video coaching sessions) to go deeper into the content they thought they needed most without feeling rushed. However, the desire for the pace to be more personalized did not impact their satisfaction with the program.

A minority of participants noted difficulty understanding or remembering the content between sessions.

By the time I got to the second module, I would forget about the first one. I would forget notes I had for my coach and was often unable to write down my questions. I would forget before next time.

45-54-year-old White female

Some participants successfully prepared for coaching sessions by taking detailed notes about the content and their questions, or by reviewing the webSTAIR content immediately before meeting with their coach. This strategy was acceptable for some webSTAIR participants, but others found it tedious or “annoying” to have to refresh between sessions to avoid forgetting the content, though this was uncommonly reported.

### Theme 3: Comfort and Convenience of the Web-Based Format Made it Easier to Access Care

Participants liked the convenience of the coach-guided, web-based format, which allowed flexible scheduling and reduced travel time. The ability to meet on the web allowed veterans to engage in care they may not have otherwise engaged in due to distance or other logistical factors, such as difficulty scheduling around work or childcare obligations. A Hispanic male in his late 30s explained how and why the web-based format was more convenient:

Able to do it from home. I cannot take advantage of many mental health services at the VA; VA is 30-40 minutes from my home. I was glad I was able to take advantage of this.

In addition to the logistical benefits, participants liked that they did not have to confront crowded waiting rooms at their facility. Instead, they could participate in the program from home, where they felt most comfortable and, in some cases, where they felt most safe. A minority of participants found it more difficult to find privacy in their own homes, away from the people they lived with, and others found themselves more prone to distractions at home. According to 1 participant, a White female in her mid-forties, “…the same thing that made it nice also made it not so nice.” However, privacy issues or distractions at home were not commonly reported.

### Theme 4: Technical Issues Are Common but not Insurmountable

The majority of participants experienced some technical challenges with the video coaching sessions, and almost half experienced difficulty with the website. Participants spontaneously reported that about a third of all the technical challenges they experienced occurred only 1 or 2 times throughout the entire program. For issues that did not resolve on their own, participants were able to troubleshoot, either independently or with their coach, to complete their sessions and web-based content. In cases where coaches were able to help solve problems or troubleshoot, their guidance seemed to ameliorate the negative impact on patient satisfaction.

[Had] difficulties quite a few times, but she always made it work.

35–44-year-old Hispanic female

Technical issues included being unable to hear the other person, issues with the link to the video session, and difficulty with the video freezing or closing unexpectedly. Sometimes the issue resolved quickly on its own and occurred infrequently. Other times, the issue could be attributed to a recent update or a setting that needed to be changed on their device. Strategies that were frequently used by coaches to help troubleshoot included pivoting to the phone for audio, sending different pieces of equipment to use, and offering emotional support that someone would help them resolve the issue. A White female in her mid-forties said:

One time we [experienced video issues]; [we] just turned off the audio, [and] video was still there and [we] used phone for audio. Work around; it worked just fine.

Only 1 participant spontaneously reported having had to reschedule a session with their coach due to connection issues. More often, participants shared that they were still able to meet on the web with their coach despite issues with the video connection, in part because of their coach’s persistence.

It didn't connect twice but she worked hard to get it connected, so we still had our sessions.

35–44-year-old African American female

Participants reported a wide range of issues with the website, including trouble accessing the tools or videos, glitches such as issues with the “back” or “next” button, difficulty with the website kicking them out or freezing up, and issues with the equipment that made accessing all the content from the modules difficult. Several participants spontaneously reported that they resolved the issue with the website through independent troubleshooting, usually by closing and reopening the browser: “Sometimes the role play [exercise] wasn't working right. Closed out the browser and then it would work fine” (35-44–year-old Hispanic female). In at least 1 case, an issue with the website was resolved when the coach sent the participant a VA iPad (Apple Inc). Issues that were most easily resolved included the website or web content freezing up and issues with the “back” button not working.

More persistent issues with the website included typed responses not saving, being unable to access program materials, such as worksheets or role plays, and difficulty printing. There was only 1 case in which a participant spontaneously shared that they had to reschedule a session due to technical issues with the website and an inability to access program materials. More often, participants found workarounds, like taking screenshots instead of printing and writing down their responses.

Typing info into forms/worksheets. Happened 2 times, then I quit. I started writing it down.

45–54–year-old White female

## Discussion

### Principal Results

This study contributes to the literature on satisfaction with web-based mental health treatments by qualitatively exploring mechanisms that lead to better or worse patient experiences with technology-based mental health tools, in addition to satisfaction with the patient-provider relationship. Results from this study may help clinicians and researchers tailor interventions in a way that increases satisfaction with web-based mental health treatments. Our chief findings were as follows: (1) the presence of a coach or therapist to help guide a web-based intervention was vital for accountability and support; (2) a flexible, self-paced format enabled participants to fit the treatment into their schedule more easily and give them time to reflect on the content in between sessions; (3) participants liked the comfort and convenience of the web-based format, as it decreased travel time and opened up access to mental health services that may otherwise have been too difficult to obtain; and, (4) despite most participants experiencing technical difficulties, a common issue with web-based treatments, participants in our sample were able to troubleshoot those issues and complete the program and it did not appear to impact their overall satisfaction. Although participants generally reported positive responses to the intervention, some reported a desire to have more control over the pacing of modules based on their individual mastery of the module’s content.

### Comparison With Previous Work

Consistent with the literature, the coach-guided support contributed to the high level of satisfaction observed in this study. Participants noted the importance of the coach for their support and accountability. Coaches helped keep participants on track, knowing that they are going to discuss the module in their next session. Coaching helped participants apply the content to their own lives and made it relatable, enhancing the program’s value for patients. Consistent with previous work by this team, participants in this study often noted a high level of satisfaction with their coach, and many commented on the likeability or effectiveness of their coach [40]. Previous work suggests that engagement and efficacy in technology-based mental health interventions are often low without therapist support [41], which may be why the value of coaching was such a prevalent theme in our exploration of satisfaction in this study. Like participants in other coach-guided, internet-based studies, webSTAIR participants experienced excellent relationships with their coaches, and they were able to achieve this over video [30,42-44].

Previous studies of technology-based mental health interventions suggest that the most frequently reported reason for dissatisfaction is related to intervention pacing that moves too quickly [45]. Participants in this study often noted the benefit of the self-paced format of webSTAIR, as it allows more time and space to reflect on, practice, and absorb the content compared to standard, synchronous, evidence-based psychotherapy sessions in which participants may feel pressure to respond and process material in the moment. Additional time to review the material between professionally guided sessions contributed to the considerable satisfaction that we have observed in this study.

Participants in this study generally appreciated the comfort and convenience of the web-based format. Approximately 62% (46/74) of our participants were rural, so decreasing drive time to their provider, in some cases, may have enabled participants to receive care they would not have otherwise been able to access. About 57% (42/74) of our participants were women, and all had a history of trauma. This may have contributed to why participants felt more comfortable receiving their care in their own homes and why they appreciated avoiding crowded VHA waiting rooms. These findings echo previous studies that found similar benefits of telehealth for patients with obsessive-compulsive disorder and veterans receiving mental health care over video telehealth [29,46].

When using video telehealth, about a third of participants noted they only encountered 1-2 technical issues, which they were able to resolve with minimal interruption. Others with more significant interruptions were able to troubleshoot on their own or with their coach to eventually get connected or access the web-based material. Since this study describes the experiences of participants who completed webSTAIR, we can presume that they were able to navigate technical issues and complete the program. Given these findings, we can infer that if a technical issue is eventually resolved, the presence of the technical issue may not impact overall satisfaction. For this reason, resilience, supportive troubleshooting, and having a backup plan (eg, using the phone for sound when audio does not work) may contribute to greater satisfaction and the successful completion of internet-based interventions.

### Limitations

The primary limitation of our study is selection bias. Our interview sample consisted of participants who completed the webSTAIR program and were prescreened to have adequate access to video telehealth. These factors may result in more positive experiences with the program, as reflected in the data. The 37% (74/202) response rate may have further exacerbated selection bias and resulted in more positive feedback. These patients were more likely to have the time and motivation to participate in an interview. The experiences of those who may not have felt the program’s impact or did not feel inclined to participate in an interview were not captured. Additionally, the study was not randomized, and we do not have a control group to compare satisfaction among those who dropped out of the study. However, as a qualitative study, our aim was to provide a detailed description of participant experiences and explore how common themes across experiences may be relevant to clinicians and researchers interested in web-based mental health treatments.

### Conclusions

Our findings suggest that a combination of self-paced, web-based content and a coach-guided intervention may integrate the best of both worlds, that is, the convenience and flexibility of a web-based modality and the structure, guidance, and accountability of a traditional psychotherapy approach. Our results indicate that providers and researchers should design tech-based interventions that are sensitive to individual differences and that integrate coaching support, as participants highly value coaching support for their digital interventions. Future research should focus on the impact of satisfaction on engagement, retention, and the therapeutic benefits of web-based mental health interventions.

## Appendix

Multimedia Appendix 1 Interview questions.

## Abbreviations

PC-PTSD: Primary Care PTSD Screen
PHQ-2: 2-item Patient Health Questionnaire
PTSD: posttraumatic stress disorder
STAIR: Skills Training in Affective and Interpersonal Regulation
VA: Veterans Affairs
VHA: Veterans Health Administration
