# Supplementary material for: Patient Satisfaction With a Coach-Guided, Technology-Based Mental Health Treatment: Qualitative Interview Study and Theme Analysis
Source: JMIR Ment Health. 2024 Feb 2;11:e50977. doi: 10.2196/50977 (PMC10873794; doi:10.2196/50977)
Supplement: Multimedia Appendix 1 [file mental_v11i1e50977_app1.docx]

**Appendix I: Interview Questions**

“Overall, did the webSTAIR program meet your needs? (Met none of my needs, Met some of my needs, Met most of my needs, Met all of my needs)”

“How likely are you to try another web-based therapy program with a coach? (Not at all likely, Somewhat likely, Very likely)”

“What did you like about the webSTAIR program?

“What didn’t you like about the program?”

“Was this the right number of sessions for you? Would you have preferred more sessions or fewer sessions? (Right number of sessions for me, Would have preferred fewer sessions, Would have preferred more sessions)

“Why?”

“What was it like working through the modules on your own without the assistance of your coach?” In addition to the question above, Coach 5 participants were asked about their experience working through multiple modules independently before meeting with their coach, “What was it like going through several modules before discussing with your coach?”.

“What did you like about the webSTAIR program?”

“What didn’t you like about the program?”

“Did you experience any difficulties with the video connection to ________ (coach name)? (yes/no)”

“If yes, what were the difficulties you experienced?”

“Did you experience any difficulties with the technology for the webSTAIR website? (yes/no)”

“If yes, what were the difficulties you experienced?”
